# Analysis of sucrose addition on the physicochemical properties of blueberry wine in the main fermentation

**DOI:** 10.3389/fnut.2022.1092696

**Published:** 2023-01-19

**Authors:** Junbo Liu, Qian Wang, Liping Weng, Ligen Zou, Huiyan Jiang, Jing Qiu, Jiafei Fu

**Affiliations:** ^1^Institute of Agricultural Products Processing, Hangzhou Academy of Agricultural Sciences, Hangzhou, China; ^2^Department of Environmental Health and Food Science and Technology, Hangzhou Wanxiang Polytechnic, Hangzhou, China

**Keywords:** physicochemical properties, blueberry, wine, main fermentation, sucrose

## Abstract

**Introduction:**

Harvested blueberries can be processed into wine to extend their shelf life and increase their commercial value. In order to produce fruit wine, external sugar is often added prior to fermentation to increase the final alcohol content to a target of 8–12% (v/v) to meet consumer expectations.

**Method:**

we explore the effect of 8–14% (w/w) sucrose on the physicochemical properties of blueberry wine throughout the main fermentation process. We monitor changes of alcohol content, sugar, color, phenol, acidity, anthocyanin, and odor.

**Results and discussion:**

We notice that sucrose affects the fermentation process and physicochemical composition of the final blueberry wine by fermentation rate, fermentation color and protection of functional substances protection. Additional sucrose extends the total time of fermentation, and increases wine acidity. The color of the wine is also affected, with added sugar darkening and yellowing the final product. Interestingly, the sucrose has a protective effect on anthocyanin levels, although total anthocyanin levels are still substantially reduced following fermentation. Finally, the additional sugar increases accumulation of volatile odor components, particularly alcohols and esters, as measured by an electronic nose. We conclude that an addition of 12% sucrose produces wine with superior physicochemical properties of alcohol, anthocyanin loss and odor relative to other conditions tested and recommend this approach to commercial manufacturers.

## Introduction

Blueberries are colloquially referred to as the king of fruits and universally loved by consumers (1, 2). Blueberries are rich in carbohydrates, organic acids, anthocyanins, phenols, flavonoids and other beneficial nutrients with antioxidative (3) and immunostimulatory effects (4), which can improve hypoglycemia (5), and protect eyesight (6). However, blueberries have a limited shelf life of 3–7 days at room temperature, resulting in rapid deterioration and short retail distance (7, 8). Furthermore, blueberries with thin skin and higher flesh content are at increased risk of microbial infection (9). For these reasons, commercial harvest enterprises can incur serious losses from spoilage following a typical summer harvest. To avoid this, fresh blueberries can be fermented into wine (10), dried (11), or juiced to extend their shelf life and increase the profitability of a harvest (12).

Blueberry wine is a high-value processed product from the highly perishable blueberry fruit, which is widely consumed (13). In recent years, the production of blueberries in China has gradually increased. Blueberries have berry characteristics similar to grapes and are used for fermentation and wine production. In the Chinese market, blueberry wine is mainly dry red. Blueberry wine is produced from fresh or frozen blueberry fruit through crushing, fermentation, aging, filtration, filling and packing (14). The fermentation process produces alcohol and carbon dioxide from metabolism of sugar by wine yeast (*Saccharomyces cerevisiae*). The sugar content of blueberry is generally 80–100 g·L^−1^, and lower than grapes of 214–228 g·L^−1^ (15), the most widely-used fruit to produce wine. While 17 g·L^−1^ sugar present in blueberries should theoretically produce 1% (v/v) alcohol after fermentation, consumers generally expect an alcohol content of 8–12% (v/v). To produce the proper alcohol content, commercial producers typically supplement the crushed fruit with sucrose, an economical sugar source. However, it is unclear how this additional sucrose affects the odor and physicochemical properties during fermentation.

The fermentation process of blueberry wine can be divided into a main and secondary fermentation stage (16). During main fermentation, yeast propagate and produce ethanol (2). This dynamic stage also results in the degradation, transformation, generation of new substances and chemical byproducts. Conversely, the post fermentation process is predominantly a odor improvement stage during which lactic acid bacteria (LAB) degrade malic acid to produce lactic acid and catalyze additional chemical reactions which influence the aroma of the wine (17).

In this study, we investigate the impact of sucrose addition during the main fermentation process on the physicochemical properties of the finished blueberry wine, including the alcohol content, nutrient abundance, and odor profile. We expect these results can be used to improve commercial-scale production of blueberry wine.

## Materials and methods

### Materials

Blueberries, the variety of rabbit eye, purchased from Hangzhou Gaofeng Blueberry Planting Co., Ltd (Hangzhou, China). Yeast (*S. cerevisiae*, RW) was provided by Angel Yeast Co., Ltd (Yichang, China). Malvidin, delphinidin, cyanidin, peonidin and petunidin were purchased from Beijing Tanmo Quality Inspection Technology Co., Ltd (Beijing, China).

### Preparation of blueberry wine

Fresh or frozen blueberries were washed and crushed at a temperature no >35°C before transfer to a sterilized fermenter and heated to 35°C. Potassium metabisulfite was added to produce a final sulfur dioxide concentration of 30 mg·kg^−1^. Pectinase [≥5,000 U/mg, Novozymes (China) Biotechnology Co., Ltd] was added to a final concentration of 30 mg/kg (w/v) and allowed to react for 1.0 h. Additional sucrose, if present, was added at concentrations of 8, 10, 12, and 14% (w/w) prior to fermentation. Yeast was dissolved in 10 times of water at 30°C and activated for 10 min according to manufacturer's instructions and added at a final concentration of 200 mg·L^−1^ at 30°C. Samples were taken at 2, 4, 6, 8, and 10 days for further physicochemical analysis. All experimental groups were fermented 3 batches.

### Alcohol quantitation

A volume of 100 mL blueberry wine was removed from the fermenter and transferred into a 500 mL distillation flask. The distillate was collected in a water bath held at 20.0 ± 0.1°C for 30 min, and the alcohol content was measured using a density flask.

### Acid quantitation

The Acid content of the wine was assayed according to Chinese national standard (18). A volume of 1 mL blueberry wine was transferred to a conical flask and mixed with 100 mL water and two drops of phenolphthalein indicator solution. Then the solution was titrated to the end point with 50 mM sodium hydroxide until the color remained unchanged for 30 s. A control experiment without blueberry wine was performed in parallel.

Tartaric acid was quantified using the following formula:


(1) $$\begin{array}{l} X=C\times \frac{V1-V0}{V2}\times 75 \end{array}$$

where *X* is [Tartaric acid] (g·L^−1^), *C* is [NaOH], mol/L, *V*_1_-*V*_0_ is the difference in volume of NaOH added in the sample and control titrations, respectively (mL), and *V*_2_ is the volume of blueberry wine present in the sample titration (mL).

### Sugar quantitation

The total sugar content of the wine was assayed by volumetric analysis according to Chinese national standard (18). First, a volume of blueberry wine was diluted tenfold with pure water and heated to boiling. Then, a solution of freshly prepared Fehling's Reagents were added at a rate of 0.5 drop/s until the end point, indicated by the disappearance of color. The total consumed volume of sample solution was recorded and repeated three times in parallel.

Sugar content was quantified according to the following formula:


(2) $$\begin{array}{l} X=\frac{F\times V2}{V1\times V3}\times 1000 \end{array}$$

where *X* is the total sugar content of blueberry wine (g/L), *F* represents the total oxidative potential of Fehling's reagents (*g* of glucose), *V*_1_ is the volume of blueberry wine sample absorbed (mL), *V*_2_ is the volume of blueberry wine sample diluted, mL, and *V*_3_ is the volume of blueberry wine sample consumed (mL).

### Colorimetric analysis

Colorimetric analysis of clarified blueberry wine was performed using a colorimeter in transmittance mode (Hunter Lab, USA). The color of each sample was quantified according to the Hunter *L, a, b* color scale which uses the following parameters: L^*^ represents brightness (where 0 is dark, 100 is light), a^*^ represents the degree of red and green (–a^*^ is green, +a^*^ is red), and b^*^ represents the degree of yellow and blue (–b^*^ is blue, +b^*^ is yellow).

### Phenol quantitation

Phenol content determination were assayed according to Martín-Gomez et al. (19)'s analytical method. A volume of 1 mL blueberry wine was diluted fivefold, and 1 mL of this diluted sample was mixed with 1 mL Folin's Phenol Reagent and 5 mL of water. After 3 min, 3 mL 75 g/L sodium bicarbonate was added and the mixture was heated to 50°C for 10 min. The A_647_ was measured using a spectrophotometer, and used to calculate total phenol content using a standard curve generated with a gallic acid standard.

### Anthocyanin characterization

Anthocyanins were characterized using Liquid chromatography tandem mass spectrometry (LC-MS/MS) (waters, Massachusetts, USA) following ultrasonic extraction at 100 w for 10 min. First, 5 g crushed blueberry mash was transferred into a 25 mL colorimetric tube, combined with two volumes of absolute ethanol, one volume of hydrochloric acid, and one volume of water, and then heated in a boiling water bath for 1 h. The sample was then sonicated for 30 min, and centrifuged at 4,000 r/min. The supernatant was collected, filtered through 0.22 μm filter, and analyzed using LC-MS under the following conditions.

Chromatographic column: Waters ACQUITY BEH C18 column (1.7 um, 2.1 × 100 mm); Column temperature: 40°C; Injection volume: 2 μL; Flow rate: 0.5 mL/min. Mobile phase: 0.1% formic acid (A) and 100% acetonitrile (B); Gradient elution procedure: 0–1.1 min, 5–10% B; 1.1–5.5 min, 10–50% B; 5.5–6.9 min, 50–5% B; 6.9–7.5 min, 5% B, balance for 1 min.

Ion source: electric spray (ESI+); Detection mode: MRM; Capillary voltage: 1.0 kV; Ion source temperature: 150°C; Cone gas flow rate: 50 L/h; Solvent gas temperature: 500°C; Solvent gas flow rate: 1,000 L/h; Collision gas (argon): 0.15 mL/min; Dwell time: 0.02 s.

Malvidin, delphinidin, cyanidin, peonidin, and petunidin were directly injected as references.

### Electronic nose analysis

A solution of 15 mL blueberry wine was collected into centrifuge tube. A 2 mL injection tube was used absorb volatile gases from the wine and inject into the electronic nose system (Zhejiang Gongshang University, China). The system is composed of an air pump, air chamber, sensor array, signal acquisition system and pattern recognition algorithm. The sensor array is composed of fourteen gas sensitive metal oxide sensors, as shown in Table 1, where each sensor is sensitive to one or more types of gases. When the volatile gases contact the sensor, the conductivity (*G*) of the sensor changes relative to the initial conductivity (*G*_0_), and the ratio *G*/*G*_0_ represents the total response. Each sample is tested six times.

**Table 1:** Detection of flavor component corresponding to sensors.

**Sensor No**.	**Sensitive gas**
S1	Ammonia, amines
S2	Hydrogen sulfide and sulfide
S3	Hydrogen
S4	Alcohol, organic solvent
S5	Toluene, acetone, ethanol, formaldehyde, hydrogen and other organic vapors
S6	Methane, biogas and natural gas
S7	Methane, propane, isobutane, natural gas, liquefied gas
S8	Smoke, cooking smell, VOC, ammonia, hydrogen sulfide and alcohol of cigarettes
S9	Butane, propane, methane, liquefied gas, natural gas, coal gas
S10	LPG, combustible gas, propane, butane
S11	Propane, smoke and combustible gas
S12	Carbon monoxide, ethanol, organic solvents, other volatile gas
S13	Smoke, cooking odor, hydrogen, carbon monoxide, air pollutants
S14	Methane, natural gas

### Statistical analysis

All data were shown as mean ± standard deviation. Each test was repeated three times. Statistical analysis was performed using EXCEL (Microsoft, USA) and SPSS version 24 (IBM, USA). Graph Pad Prism 8.3.0 (GraphPad Software, USA) was used to graph. One-way ANOVA was used to analyze the anthocyanins content data with Duncan's test, and means were considered significantly different if *p* < 0.05.

## Results and discussion

### Alcohol content

During fermentation, sugars present in the blueberry mash are gradually converted into ethanol, and alcohol content is the key indicator for evaluating the quality of blueberry wine. Fermentation begins with the growth stage ~1–2 days following addition of yeast, during which temperature and sugar content have the greatest influence on the fermentation rate (20). Then, the fermentation enters the stable stage where the total concentration of sugar reaches 5 g·L^−1^, indicating that main fermentation is complete. The main fermentation phase is usually completed in a week (21).

**Figure 1:**
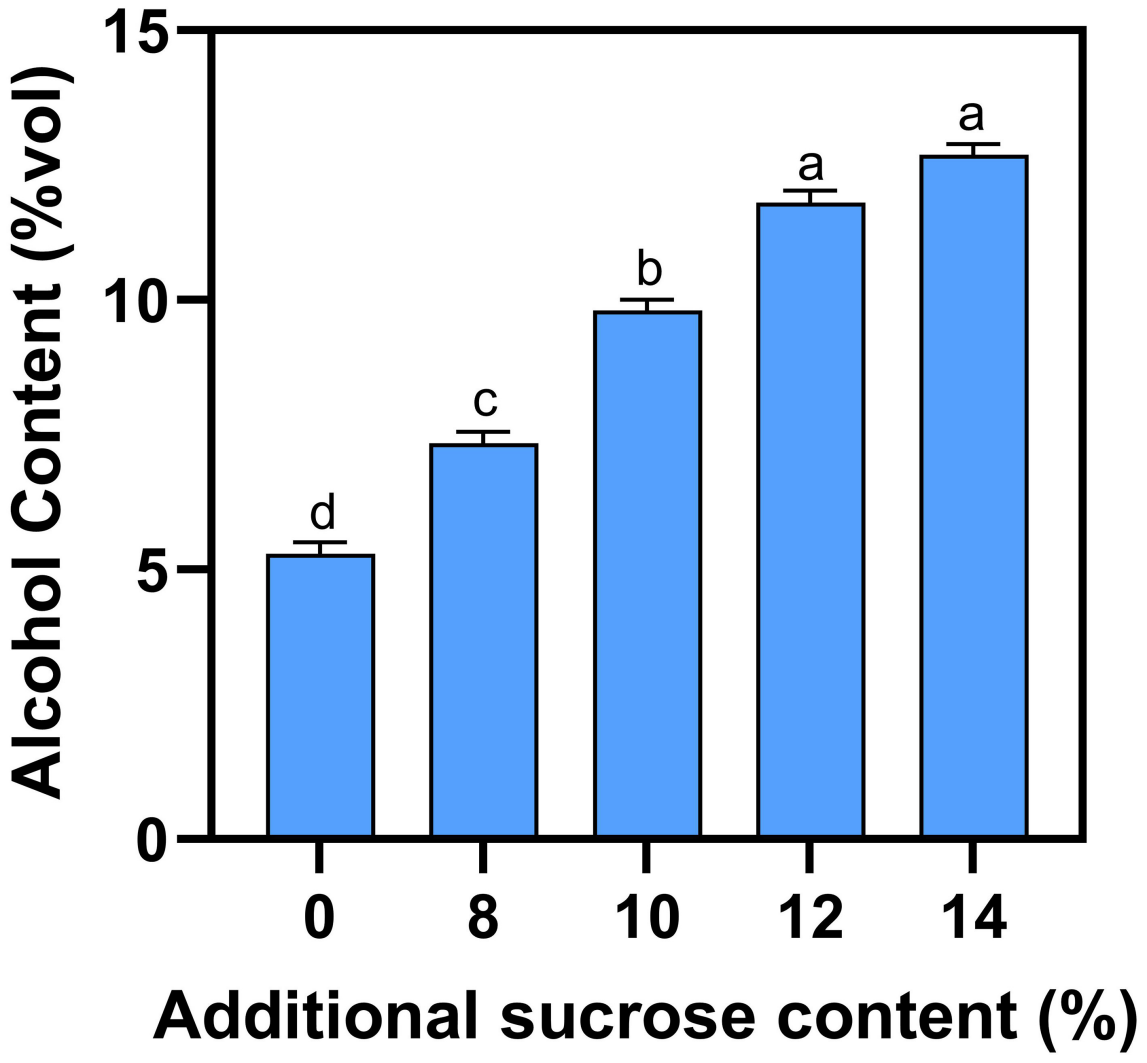
Effect of additional sucrose on alcohol content. Different superscript letters in the graph are significantly different (*P* < 0.05).

Due to the complexity of the fermentation process, the natural sugar present in the mash is usually not entirely converted into alcohol. For example, although the sugar content of blueberry fruit is 86 g·L^−1^, blueberry wine fermented without supplemental sugar only produces a final alcohol concentration of 4.5% (Figure 1). Alcohol concentration is gradually increased as external sugar is added, with a sugar content of 12% producing a final alcohol concentration of 11.7%. However, increasing sugar content from 12 to 14% does not lead to a significant increase in alcohol concentration, because yeast vitality is inhibited at high alcohol concentrations. Considering demand of consumers and fermentation capacity, 12% sugar content is recommended to obtain more acceptable alcohol.

### Acid content

**Figure 2:**
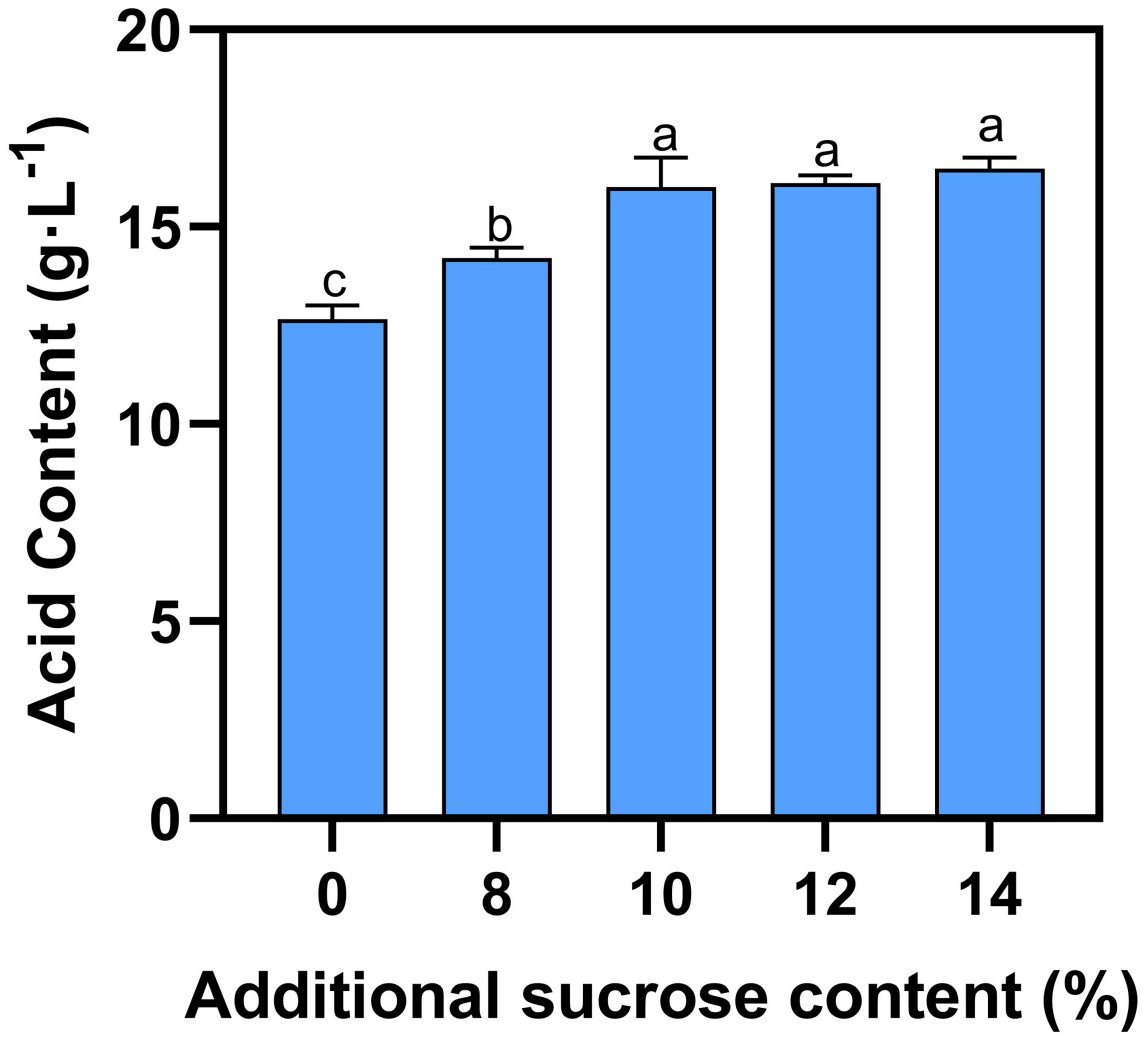
Effect of additional sucrose on acid content. Different superscript letters in the graph are significantly different (*P* < 0.05).

Pyruvic acid, acetic acid, lactic acid and other metabolites are produced by yeast during the fermentation process (22). Generally, potassium bicarbonate is added to reduce the acidity of blueberry wine, as high acidity is undesirable (14). Fermentation of natural blueberry wine without supplemental sugar yields 12.6 g·L^−1^ total acid (Figure 2). As external sugar is added during fermentation, the total acid content rises slightly to 14.2–16.5 g·L^−1^ at 8–10% sucrose, respectively. However, addition of sucrose beyond 10% does not significantly raise acidity.

### Sugar content

**Figure 3:**
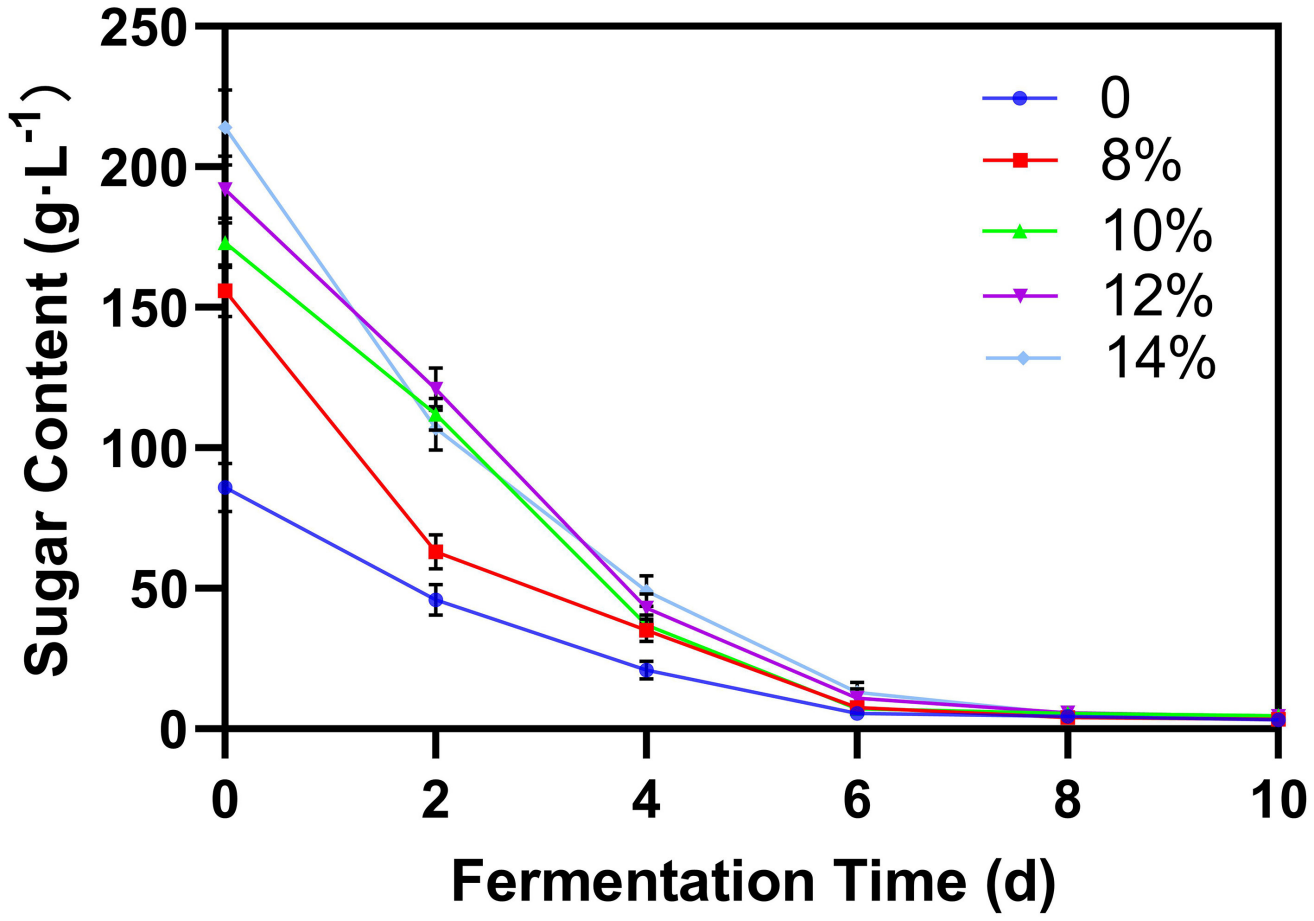
Effect of additional sucrose on sugar content during main fermentation.

Fermentation is mainly the process of decomposing sugar and producing alcohol. Therefore, monitoring the sugar content can indicate the fermentation process. For blueberry wine with external sugar concentration < 12% (w/w), the sugar content rapidly decreased to 10 g/L after 6 days and to 5 g/L after 8 days (Figure 3). For blueberry wine with 12 and 14% sugar, this trend was delayed by 2 days. However, in all cases, the fermentation process finishes within 10 days. Total sugar content serves as a proxy for the dynamic blueberry fermentation process (23), and once the post fermentation stage begins, operations such as blueberry residue filtration could be implemented to save time and avoid negative impact of residual microorganisms in wine puree on odor and quality. It is necessary to dynamically monitor the change of sugar content during wine fermentation (24).

### Color

**Figure 4:**
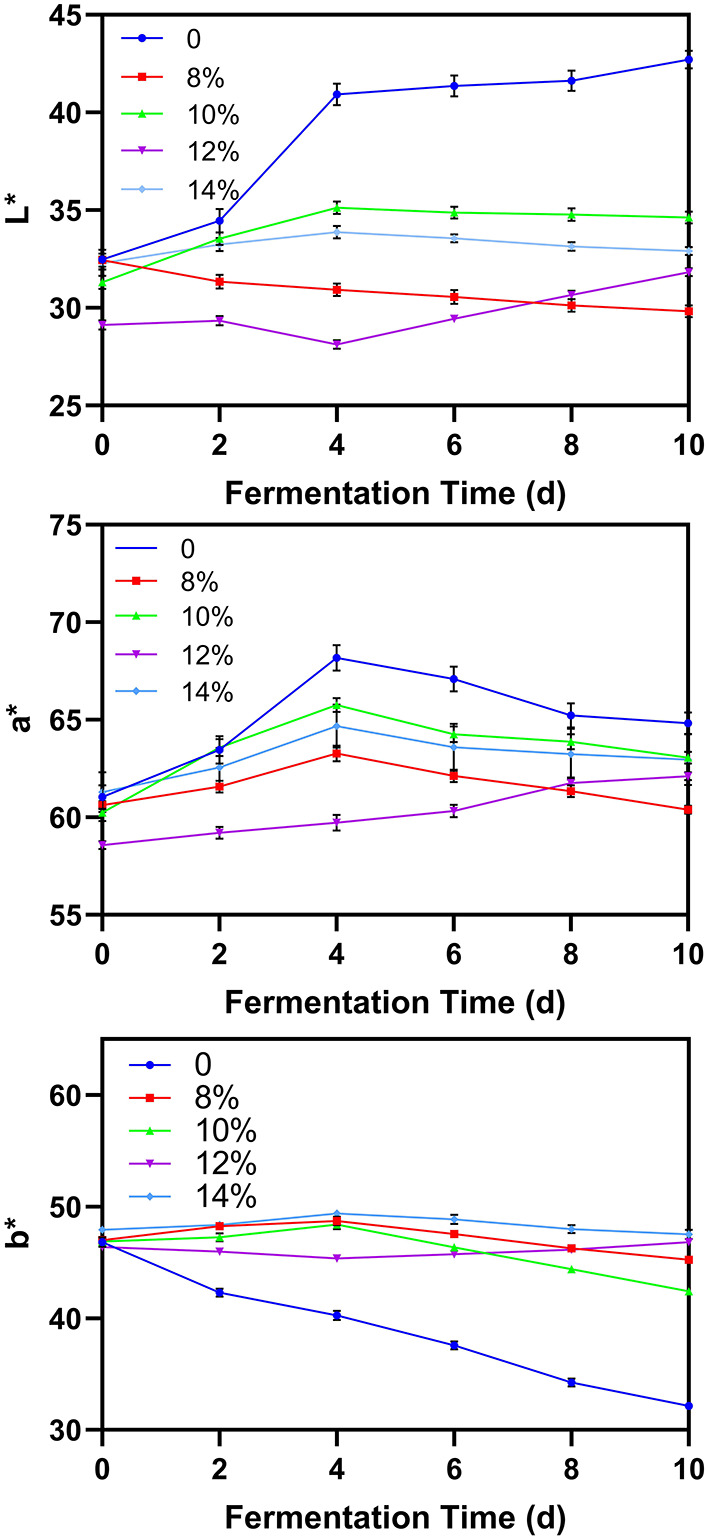
Effect of additional sucrose on L*, a*, and b* during main fermentation.

The chromaticity parameters L^*^, a^*^ and b^*^ can be used to infer the quality of blueberry wine throughout fermentation (13). In blueberry wine with added sucrose, the L^*^value, representing brightness, is lower than in wine without additional sugar, whereas the b^*^ value representing yellow color is higher (Figure 4). This may be a result the added sucrose producing a cumulative increase in fermentation products which darken the wine and alter its color. Interestingly, the a^*^ values which represent red color first rose and then slowly decreased during the fermentation process. The initial increase may be a result of red-colored pigments in residual blueberry peel dissolving into solution, whereas the subsequent decrease may result from yeast metabolizing these compounds as fermentation continues. This result is consistent with current literature, with Li et al. also showing that the a^*^ of blueberry wine increased first during fermentation and then decreased.

### Phenol content

**Figure 5:**
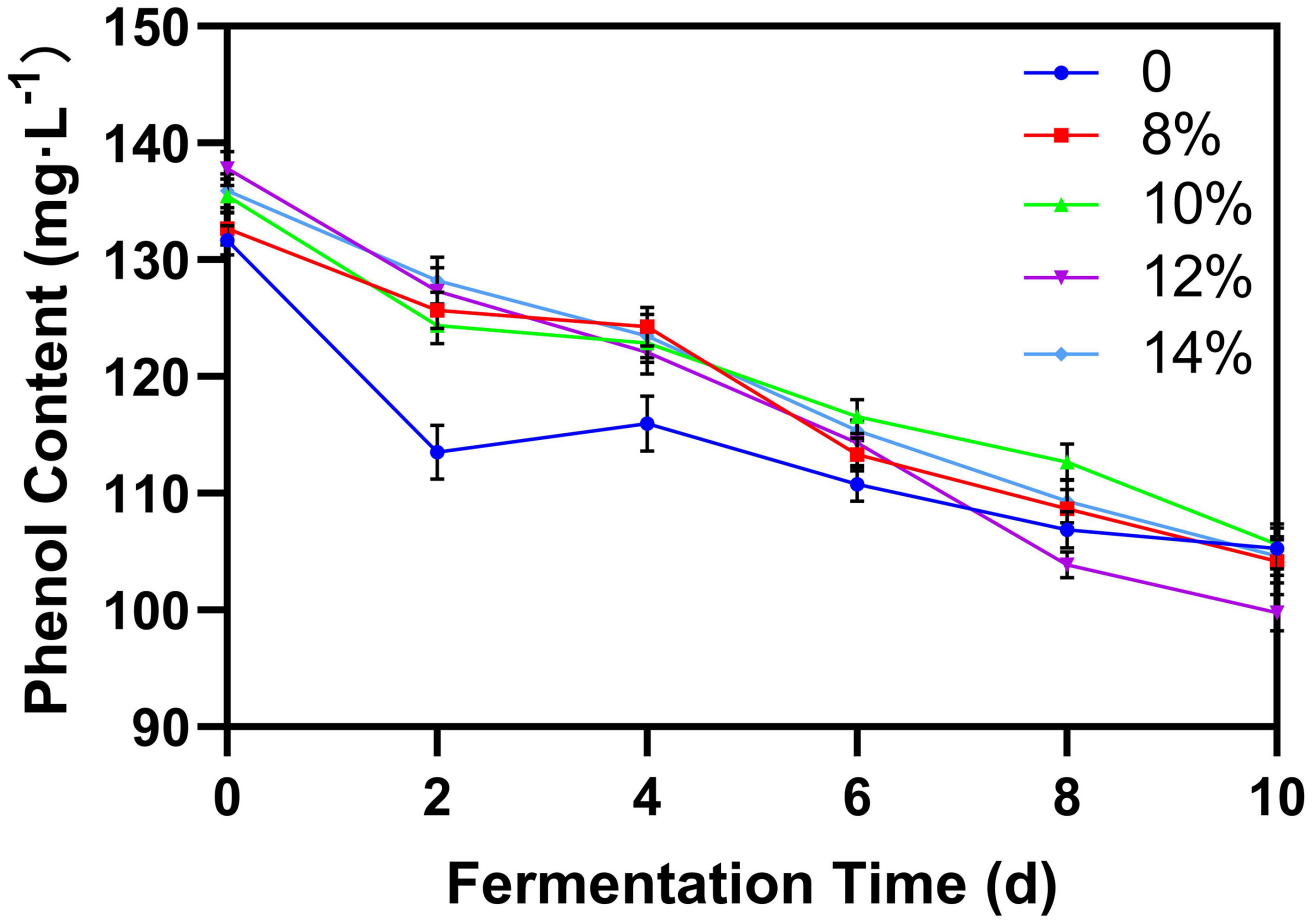
Effect of additional sucrose on phenol content during main fermentation.

Phenols are the key substances determining the biological activity of blueberries (19, 25). The composition of phenolic substances is relatively complex. Figure 5 shows the change in phenol concentration during the blueberry fermentation process. In all cases, phenol concentration declined slowly as fermentation progressed, likely due to the metabolism of phenolic substances by the yeast or the reaction with other byproducts of fermentation (26). Although phenol concentration decreased for all groups, blueberry wines with added sucrose showed higher phenol levels early in the fermentation process, suggesting that additional sugar can prevent the degradation of phenols. However, by the end of fermentation, after the external sucrose was fully converted into ethanol, there was no significant difference in the phenol content between samples.

### Anthocyanin characterization

**Figure 6:**
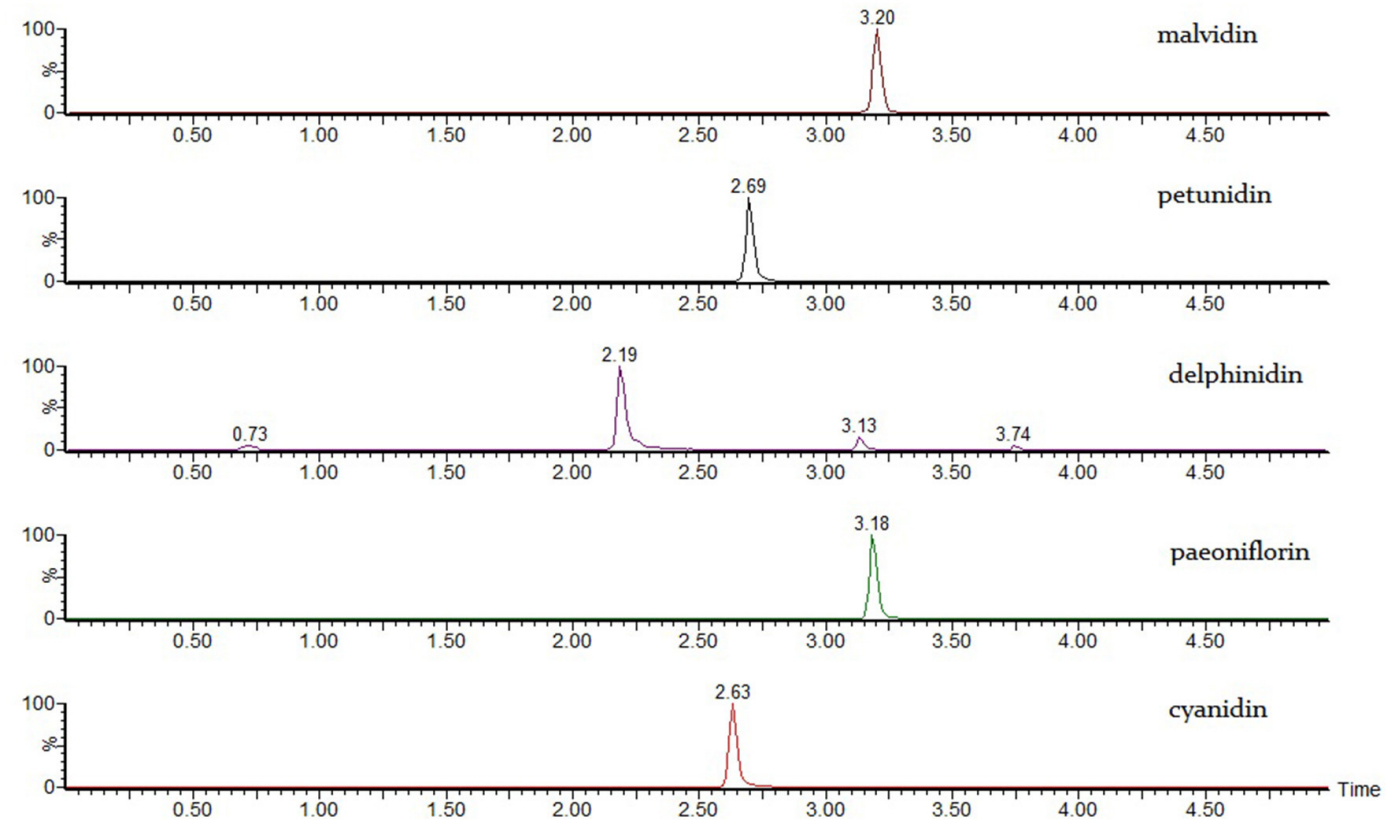
Mass spectrogram of malvidin, petunidin, delphinidin, paeoniflorin, and cyanidin.

Anthocyanins are the most concerned components in blueberry processing because of antioxidant activity and health benefits (27). Anthocyanins are susceptible to several processing and storage conditions, such as pH condition, temperature and light exposure, etc. (28) and the degradation and loss of anthocyanin are inevitable during the fermentation solution environment (28). Anthocyanins are composed of cyanidin, delphinidin, malvidin, petunidin and peonidin and glycosidic groups (29), and can be deglycosylated into their corresponding aglycone structure by heating in acidic environment. The resulting cyanidin, delphinidin, malvidin, petunidin, and peonidin correspond to their native anthocyanin forms and can be quantitatively analyzed using LC-MS/MS (30). Because blueberries contain a wide variety of anthocyanins, it can be difficult to find appropriate reference samples to accurately quantify them (31). Furthermore, the retention time of aglycones are similar on HPLC, resulting in poor resolution and inaccurate quantification. However, liquid chromatography coupled with tandem mass spectrometry (LC-MS/MS) can avoid the disadvantages of HPLC by accurately determining and quantifying both the parent ions and the daughter ions of the substance resulting from a specific collision energy. Importantly, the cyanidin, delphinidin, malvidin, petunidin, and peonidin references were well resolved using this technique (Figure 6).

**Figure 7:**
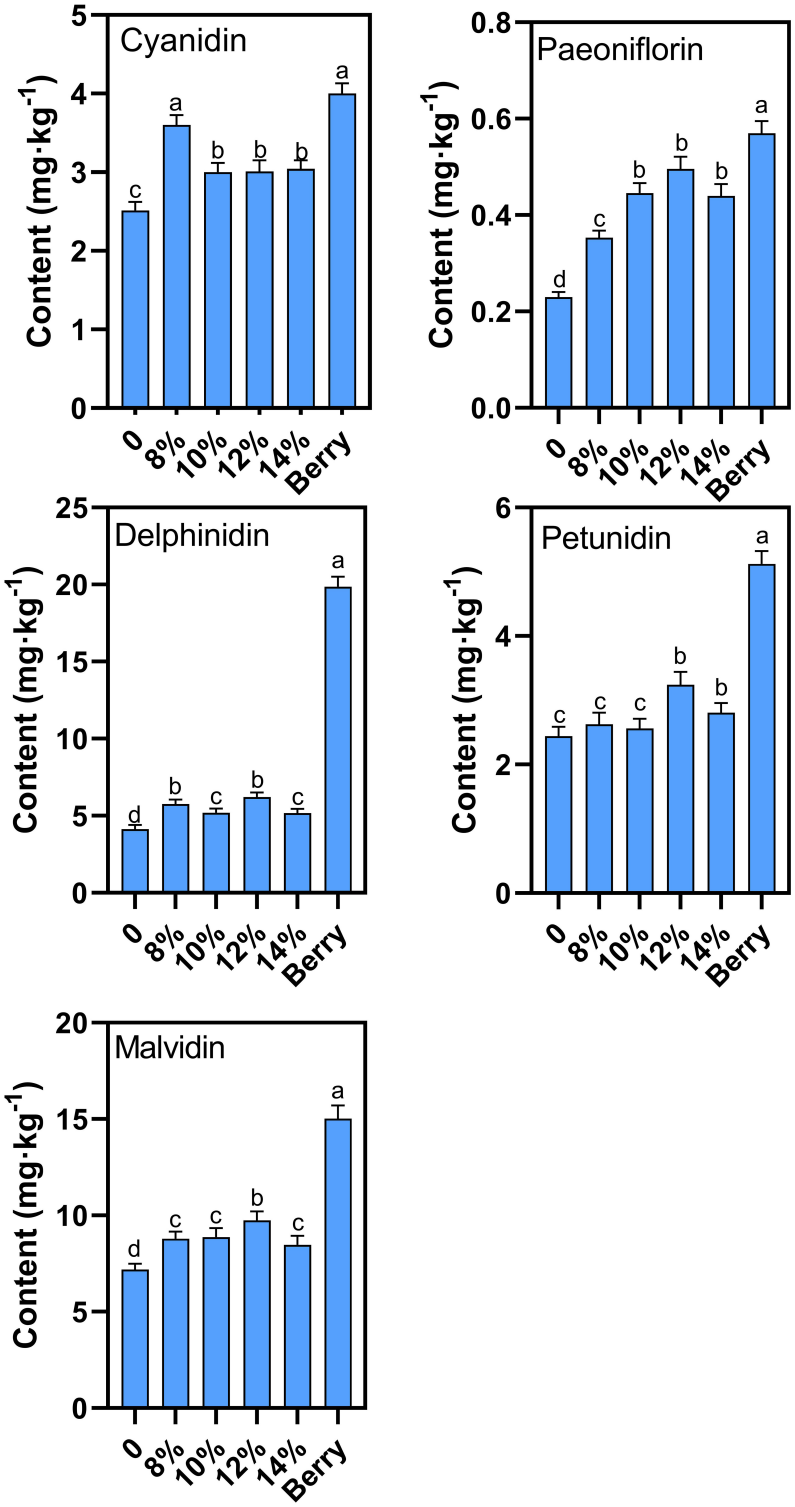
Effect of additional sucrose on anthocyanins after main fermentation. Different superscript letters in the graph are significantly different (*P* < 0.05).

Delphinidin and malvidin are the two most abundant anthocyanins in blueberries, which were both lost to varying degrees after the main fermentation (Figure 7), consistent with previous literature reports (23). Groups with added sucrose show significantly higher anthocyanin levels suggesting that additional sucrose can improve anthocyanin stability during fermentation. A similar protective function resulting from mannoproteins has also been demonstrated in the literature (17). The groups with 12% external sucrose showed the smallest loss in overall anthocyanin concentration during fermentation, with a anthocyanin content of 51.9% relative to levels prior to fermentation. Within the analyzed anthocyanin classes, the degree of degradation varied between classes. Delphinidin and malvidin levels showed the greatest loss during fermentation, with delphinidin levels 31.3% of their original concentration and malvidin levels 64.8% of their original concentration. Therefore, 12% sugar addition concentration was recommended because the favored anthocyanin loss was minimal.

### Electronic nose analysis

**Figure 8:**
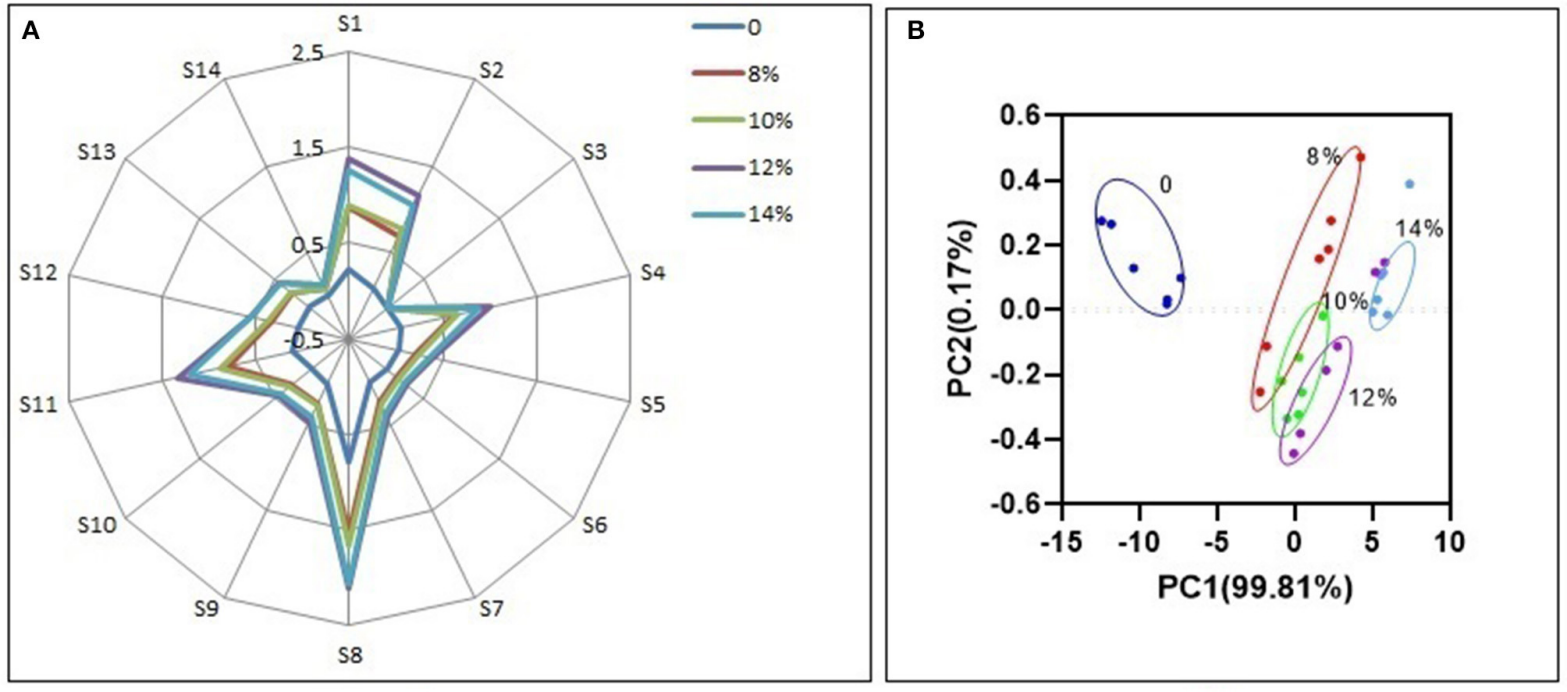
Odor response value of electronic nose **(A)** and PCA **(B)**.

It is possible to characterize the odor profile of wine using an electronic nose, which represent fourteen kinds of aromatic substances produced during blueberry wine fermentation (32, 33). For all samples, sensors S8, S1, S2, and S4 have the highest response (Figure 8A), and represent volatile alcohols, aromatic esters, sulfides and amines, respectively, which together constitute the characteristic odor components of blueberry wine. Alcohols, esters and amines are metabolites naturally produced by yeast during fermentation, while sulfides originate from the addition of potassium metabisulfite to inhibit bacteria growth. Additional sucrose does not change the basic odor composition of blueberry wine, reflected by the similar shape of the electronic nose odor profile between all samples. Compared with the experimental group without added sucrose, the added sucrose groups had similar odor composition, which indicates that the added sucrose produces specific odor components. However, additional sucrose enhances the response of each odor substance compared with the control group, especially the group of 12%. Although sucrose is metabolized to produce alcohol in the fermentation process, its metabolism also promotes the accumulation of odorful substances like esters, indicated by the increased response of sensor S8.

Although the odor composition is similar, different groups of blueberry wine can be distinguished by principal component analysis (PCA) of electronic nose data (Figure 8B). Principal factor 1 (PC1) explained 99.81% of the variance in the data and principal factor 2 (PC2) explained 0.17% of the variance. The DI value was 84.39%, above the required 80% cutoff (34), indicating PCA can effectively discriminate electronic nose profiles with good repeatability.

## Conclusion

The physicochemical properties blueberry wine and the effect of external sucrose during main fermentation process of were analyzed by measuring alcohol content, sugar, color, phenol, anthocyanins, acid, and odor. Although yeast strain and sulfur dioxide were not investigated as part of this study, these also impact the physicochemical properties of blueberry wine and warrant further investigation. Additional sucrose extends the fermentation time and increases the acid content relative to natural blueberry wine. The extra sugar also alters the color profile of the wine, by lowering L^*^ and raising b^*^ relative to natural blueberry wine. Although total anthocyanin levels are reduced throughout the fermentation process, addition of sugar produced a protective effect on delphinidin, cyanidin, paeoniflorin, and petunidin, and especially malvidin. Finally, additional sugar enhanced volatile odor components in blueberry wine, particularly of alcohols and esters, as measured by an electronic nose. Taken together, these data recommend addition of 12% sucrose in commercial preparation of blueberry wine especially for physicochemical properties of alcohol, anthocyanin loss and odor.

## Data availability statement

The original contributions presented in the study are included in the article/supplementary material, further inquiries can be directed to the corresponding authors.

## Author contributions

JL and JF designed and conceived the study. LW, JQ, and HJ performed the experiments. QW analyzed the data and drafted the manuscript. JL, QW, LZ, and LW contributed to the writing of the manuscript. LZ provided the funding and resources. All authors revised and approved the submitted version of the manuscript.
